# Substance Use Is Common in Elder Abuse Cases: Qualitative Findings From a Multi-Disciplinary Team

**DOI:** 10.1093/geroni/igaa057.1442

**Published:** 2020-12-16

**Authors:** Seyed Parham Khalili, Alyssa Elman, Sunday Clark, Yuhua Bao, Yiye Zhang, Philip Jeng, Katherine Wen, Tony Rosen

**Affiliations:** 1 Rutgers University, Philadelphia, Pennsylvania, United States; 2 Weill Cornell Medicine, New York, New York, United States; 3 Weill Cornell Medical College, New York, New York, United States; 4 Cornell University, Ithaca, New York, United States; 5 Weill Cornell Medicine / NewYork-Presbyterian Hospital, New York, New York, United States

## Abstract

Elder abuse is mistreatment of an older adult by a caregiver or another person in a position with an expectation of trust. Adversely affecting as many as 10% of community-dwelling older adults in the US, it may include physical abuse, sexual abuse, psychological abuse, financial exploitation and neglect. Mental illness and substance use by caregivers, family members and victims themselves have been described as risk factors for multiple forms of elder abuse in prior cross-sectional analyses but the impact on these cases is poorly understood. To explore this association we conducted a focus group using a semi-structured format involving an inter-disciplinary group of elder abuse professionals that are part of the New York City Elder Abuse Center enhanced multi-disciplinary team (EMDT) in Staten Island, New York. Focus group participants reported that opioid, cocaine, cannabis and alcohol use is common among perpetrators of elder abuse, especially in cases of financial mistreatment, verbal and physical abuse. Other potential consequences included eviction of the older adult victim, co-dependency and involvement of the older adult in the procurement of illicit substances, and substance use by the older adult. Respondents specifically expressed concerns that the opioid epidemic, including rising heroin use, may be changing the frequency and nature of elder abuse, and that case investigations offer an opportunity to facilitate referrals for formal substance use disorder assessment and treatment. Future work includes additional focus groups and quantitative analysis to clarify the intersection between substance use and elder abuse and inform intervention and prevention strategies.

